# Comparative Analysis of miRNA Expression Profiles between Heat-Tolerant and Heat-Sensitive Genotypes of Flowering Chinese Cabbage Under Heat Stress Using High-Throughput Sequencing

**DOI:** 10.3390/genes11030264

**Published:** 2020-02-28

**Authors:** Waqas Ahmed, Ronghua Li, Yanshi Xia, Guihua Bai, Kadambot H. M. Siddique, Hua Zhang, Yansong Zheng, Xinquan Yang, Peiguo Guo

**Affiliations:** 1 International Crop Research Center for Stress Resistance, College of Life Sciences, Guangzhou University, Guangzhou 510006, China; ahmed18@gzhu.edu.cn (W.A.); ronghua@gzhu.edu.cn (R.L.); xiayanshi922@163.com (Y.X.); yangxq@gzhu.edu.cn (X.Y.); 2 United States Department of Agriculture–Agricultural Research Service, Hard Winter Wheat Genetics Research Unit, Manhattan, KS 66506, USA; gbai@ksu.edu; 3 The UWA Institute of Agriculture and School of Agriculture & Environment, The University of Western Australia, LB 5005, Perth WA 6001, Australia; kadambot.siddique@uwa.edu.au; 4 Guangzhou Academy of Agricultural Sciences, Guangzhou 510308, China; nkyzhanghua@gz.gov.cn (H.Z.); gznky@gz.gov.cn (Y.Z.)

**Keywords:** flowering Chinese cabbage, miRNA, heat response, high-throughput sequencing

## Abstract

Heat stress disturbs cellular homeostasis, thus usually impairs yield of flowering Chinese cabbage (*Brassica campestris* L. ssp. *chinensis* var. *utilis* Tsen et Lee). MicroRNAs (miRNAs) play a significant role in plant responses to different stresses by modulating gene expression at the post-transcriptional level. However, the roles that miRNAs and their target genes may play in heat tolerance of flowering Chinese cabbage remain poorly characterized. The current study sequenced six small RNA libraries generated from leaf tissues of flowering Chinese cabbage collected at 0, 6, and 12 h after 38 °C heat treatment, and identified 49 putative novel miRNAs and 43 known miRNAs that differentially expressed between heat-tolerant and heat-sensitive flowering Chinese cabbage. Among them, 14 novel and nine known miRNAs differentially expressed only in the heat-tolerant genotype under heat-stress, therefore, their target genes including disease resistance protein TAO1-like, *RPS6,* reticuline oxidase-like protein, etc. might play important roles in enhancing heat-tolerance. Gene Ontology (GO) analysis revealed that targets of these differentially expressed miRNAs may play key roles in responses to temperature stimulus, cell part, cellular process, cell, membrane, biological regulation, binding, and catalytic activities. Furthermore, Kyoto Encyclopedia of Genes and Genomes (KEGG) pathway analysis identified their important functions in signal transduction, environmental adaptation, global and overview maps, as well as in stress adaptation and in MAPK signaling pathways such as cell death. These findings provide insight into the functions of the miRNAs in heat stress tolerance of flowering Chinese cabbage.

## 1. Introduction

Flowering Chinese cabbage (*Brassica campestris* L. ssp. *chinensis* var. *utilis* Tsen et Lee) belongs to the Brassicaceae family and is mainly grown and consumed in southern China [1]. This vegetable crop is valuable for human diet due to its high soluble fiber, favorable taste, richness in vitamin C, and other nutrients. However, environmental stresses and climate changes have decreased the productivity and quality of this crop in recent years [2,3]. Due to the continuous increase in atmospheric temperature, heat stress is becoming a key-limiting factor for crop productivity around the globe by negatively affecting reproduction, physiological processes, adaptation, and development of many crops, thus is responsible for extensive agricultural losses [4,5]. Furthermore, heat stress affects protein synthesis, causes cell membrane damage, inactivates several key enzymes and affects cell division [6]. Heat-stress transcription factors limit the gathering of heat-shock proteins that play a significant role in plant heat-stress responses [7]. Flowering Chinese cabbage has to face the heat stress, which not only affects cellular homeostasis and plant growth but also leads to yield reductions and even plant death [8,9]. The average temperature for efficient growth of flowering Chinese cabbage is 22 °C. Head formation of flowering Chinese cabbage is sensitive to high temperature, which is irreversible, and therefore responsible for cabbage quality and yield decline in summer [10]. To survive high temperatures, plants have established various molecular and physiological mechanisms to respond and adapt to the harsh environments. Precise regulation of gene expression at the transcriptional and post-transcriptional levels is a complex process that is important to the orchestration of various plant responses to heat-stress.

Plant endogenous small non-coding RNAs (ncRNAs) can be divided into four classes: repeat-associated small interfering RNAs (siRNAs), trans-acting siRNAs, microRNAs (miRNAs), and natural antisense transcripts siRNAs, and they all play critical roles in response to different biotic and abiotic stresses. These ncRNAs use several molecular mechanisms to orchestrate the key regulatory roles, such as translation, modulation of RNA stability, and transcriptional and post-transcriptional regulation of gene expression [11]. miRNAs have become a research hotspot due to their ability to repress gene translation or target mRNA degradation to effectively control gene expression at the post-transcriptional level [12]. Numerous evidence points to the critical role of miRNAs in the modulation of important processes such as responses to environmental stresses, vegetative phase change, floral organ identity and flowering time, nutrient homeostasis, and leaf development [13]. Recently, a high-throughput sequencing approach has been used in predicting several conserved and novel miRNAs with significant functions in the plant responses to heat-stress [14,15,16]. Several databases and websites are available for analyzing and storing miRNA information. miRBase is one of the main miRNA sequence repositories and provides exact confidence levels for searching deep sequencing information with precise expression patterns [17]. Genome-wide studies demonstrated that miRNAs miR160, miR827, miR168, miR159, miR166, miR156, and miR169 significantly regulated the responses of *Brassica* plants to heat stress [11,18]. Additionally, bra-miR5726, bra-miR5714, bra-miR1885b.3, and bra-miR5716 were induced in *B. rapa* by heat stress [18]. Using high-throughput sequencing, 24 novel and 20 known differentially expressed miRNAs were identified between heat-treated heat-sensitive (HS) and heat-tolerant (HT) *Brassica oleracea* L. var *italic* genotypes [11]. Numerous miRNAs responsive to heat stress have been identified and characterized in *Brassica* spp., including *B. rapa* ssp. *chinensis* [19] and *B. juncea* [20]. However, to the best of our knowledge, limited information is available for heat-responsive miRNAs in flowering Chinese cabbage. Investigation of the functions of miRNAs under heat stress will enhance our understanding of the molecular mechanisms associated with heat tolerance that can be used in genetic improvement and production management of flowering Chinese cabbage.

Our previous work reported expressed sequence tag-simple sequence repeat (EST-SSR) markers derived from HT (Sijiu-19 and Youlv 501) and HS (3T-6 and Liuye 50) genotypes in flowering Chinese cabbage responsive to high temperatures [1]. We also identified 41 conserved and 18 novel miRNAs from small RNA (sRNA) libraries using the HT genotype, Youlv 501, after heat treatment by high-throughput sequencing [21]. Here, we performed a comparative study to identify conserved and novel miRNAs from HT (Sijiu-19) and HS (Liuye 50) genotypes at 0, 6, and 12 h of heat treatments and found that the potential targets of the differentially expressed miRNAs under the heat stress conditions were mainly involved in the regulation of biological and cellular processes, as well as catalytic and binding activities. The comparative study identified differentially expressed miRNAs and their involvements in controlling heat tolerance in flowering Chinese cabbage.

## 2. Materials and Methods

### 2.1. Plant Materials, Growth Conditions, and Total RNA Isolation

Two genotypes of flowering Chinese cabbage (*Brassica campestris* L. ssp. *chinensis* var. *utilis* Tsen et Lee), Sijiu-19 (HT) and Liuye 50 (HS), were grown in a growth chamber at Guangzhou University at 28/22 °C for 14/10 h (day/night). Plants at the five-leaf stage were transferred into another growth chamber at 38 /29 °C (14/10 h) for heat treatments. Samples were collected from the fully expanded upper leaves of HT and HS plants after 0 (control), 6 and 12 h of heat treatments. Collected tissues were flash-frozen immediately in liquid nitrogen, and then stored at 80 °C until RNA isolation [22,23]. Trizol RNA extraction kit (Invitrogen, Waltham, MA, USA) was used for total RNA isolation following the manufacturer’s protocol. 

### 2.2. Construction and Sequencing sRNA Libraries

RNA was isolated from three biological replicates at each time point and then three RNA samples per treatment were combined into one tube in an equal amount for library construction. All the cDNA libraries were constructed using TruSeq Small RNA Preparation Kit following the manufacturer’s protocol (Illumina, San Diego, CA, USA). In brief, RNA 3′- and RNA 5′-adapters were ligated to total RNA, cDNA constructs were created using reverse transcription after PCR, and then samples of small cDNA fragments of different lengths (18–30 nt) were run on 6% denaturing polyacrylamide gel by electrophoresis [18,24,25]. The final cDNA libraries were sequenced using Illumina HiSeq at the Beijing Genomics Institute (BGI, Shenzhen, China).

### 2.3. Identification of Conserved and Novel miRNAs

After removing poly-A tags, no-insert tags, adapter sequences, oversized insertion tags, 5’-primer contaminants, and small tags (sequences beyond 15–30 nucleotides or without 3’ primers), remaining sequence reads were further analyzed using the Bowtie2 web program to determine the length distribution of the sRNAs by mapping the clean reads to other sRNA databases and to the reference genome [26]. 

The unique sRNAs were aligned to known ncRNAs in the Rfam database (http://www.sanger.ac.uk/science/tools/rfam) to remove snoRNA, rRNA, snRNA, tRNA, and scRNA using NCBI BLASTN. Perfectly matched reads were excluded from further analysis; and remaining sequences were compared to *Brassica* database (http://brassicadb.org/brad/) to determine mismatched and matched sequences. These reads with no more than three mismatched nucleotides were considered as conserved miRNA candidates, whereas those with more than three unmatched sequences were considered as putative novel miRNA. miRBase software [17] was used for further prediction of the novel miRNAs.

### 2.4. Analysis of Differentially Expressed miRNAs

The levels of miRNA expression were compared between HT and HS genotypes after the heat treatments to determine differentially expressed miRNAs in flowering Chinese cabbage. To predict heat tolerance associated miRNAs in flowering Chinese cabbage, the fold-change of miRNA was determined as the ratio of miRNA expressions between HT and HS lines. The false discovery rate (FDR) was adjusted by analyzing significant *p*-value thresholds in different tests [18]. The normalized miRNA expression level was used to determine the fold changes (log_2_ ratio) of miRNA expression in each sample. To avoid calculation error, miRNA expression level was normalized and converted to transcripts per million (TPM) from 0 to 0.01 in all libraries. The minimum criteria for comparative analysis of low expression of miRNAs was adjusted as if miRNA had normalized expression of < 1 in all libraries. 

The normalization equation is as follow:

Normalized expression = actual miRNA read counts/total counts of clean reads × 10^6^.

The fold-change values and *p*-values were calculated using normalized data, and the fold-change values were used to generate a scatter plot: 

Fold-change = log_2_ (*N_2_/N_1_*).

The *p*-value was determined as follows:

$$p\left(x│y\right)={\left(\frac{N_{2}}{N_{1}}\right)}^{y}\frac{\left(x+y\right)!}{x!y!\;{\left(1+\frac{N_{2}}{N_{1}}\right)}^{\left(x+y+1\right)}}$$

$$C(y\leq \;y_{min}│x)=\sum_{y=0}^{y\leq y_{min}}p\left(y│x\right)$$

$$D(y\geq y_{max}│x)=\sum_{y\geq y_{max}}^{\infty }p\left(y│x\right)$$

where *x* and *y* denote total clean sRNA reads, while *N_1_* and *N_2_* are the normalized miRNA expression levels in the control and the treatment, respectively. 

### 2.5. Prediction of miRNA Secondary Structure 

The Zuker folding algorithm implemented in Mfold (http://mfold.rna.albany.edu/?q=mfold) was used to determine the secondary structures of miRNAs using default parameters [27]. Minimal folding free energy (MFE) and minimal free energy index (MFEI) parameters were used to differentiate the miRNAs from other sRNA sequences. Moreover, sRNA sequences sustaining Meyers guidelines were assumed as prospective miRNAs with following considerations: mature miRNAs have no more than one bulge and the bulge size is not higher than two, mismatch sequences should be less than three, and high MFEI values and high negative MFE values must be in predicted secondary structures and properly fold into stem-loop hairpin structures [28].

### 2.6. Target Prediction of Differentially Expressed miRNAs 

To identify and analyze differentially expressed miRNAs, the software packages TargetFinder [29] and TAPIR [30] were used as described earlier [31]. To achieve reliable results with a confidence interval, only common binding sites that were predicted by both tools were chosen for further analysis.

### 2.7. GO and KEGG Prediction of miRNA-Related Regulatory Pathways

Gene Ontology (GO; http://www.geneontology.org/) and Kyoto Encyclopedia of Genes and Genomes (KEGG) pathway (http://www.genome.jp/kegg/pathway.html) databases were analyzed to predict the key regulatory pathways using a corrected *p*-value (≤ 0.05) with a threshold derived from a hypergeometric test [32]. GO and KEGG analyses were classified sequence reads into different groups based on their function, including biological processes, cellular components, and molecular functions.

### 2.8. Validation of miRNAs Using RT-qPCR

To validate the sequencing data, four novel miRNAs and four conserved miRNAs—whose expressions were either down- or up-regulated—were selected randomly for RT-qPCR as previously described [33]. RT-qPCR used the same RNA samples used for sequencing that were collected from 0, 6, and 12 h of heat-treated heat-tolerant and heat-sensitive genotypes. Reverse transcription was carried out using RNA-tailing and primer-extension reverse transcription (RT)-PCR. For each quantitative real-time (qRT)-PCR, 2 μl template cDNA was mixed with 10 μl 2× SYBR Green PCR master mix (Takara Bio Inc., Kusatsu, Japan) and 5 pmol each of the forward and reverse primers in a final volume of 20 μl. The amplification program started at 95 °C for 5 min, then for 40 cycles of 95 °C for 15 s, 60 °C for 20 s, and 70 °C for 20 s, followed by a thermal denaturation step to generate the dissociation curves for verification of amplification specificity. Specific primers used in this study were listed in Table S1 with U6 as the internal control. Relative miRNA expression levels were quantified using the previously described 2^-ΔΔCT^ method [34].

### 2.9. Statistical Analysis

Statistical analysis was conducted using the statistical product and service solution (SPSS) software version 22.0 (IBM Corp., Chicago, USA). The significant differences between treatments were determined using Student’s *t*-test or Tukey’s test for multiple comparisons after one-way analysis of variance at significance level of *p* < 0.05 or *p* < 0.01. All the results were presented as means ± SEM.

## 3. Results

### 3.1. Analysis and Classification of sRNAs Sequence

Sequencing of six sRNA libraries constructed using samples from 0, 6, and 12 h of heat stress yielded 28,414,730, 24,642,112, 33,929,955 raw sequence reads from the HT genotype, and 29,468,457, 28,889,080, and 28,574,442 raw reads from the HS genotypes, respectively. The sequence data were deposited into the NCBI SRA database under the accession number PRJNA606901.

After discarding the low-quality reads that had invalid adapter, short valid length, and polyA sequences, 25,331,960, 22,632,118, 19,999,279, clean reads for the HT genotype, and 27,647,469, 26,043,571, and 26,275,173 clean reads for the HS genotype remained for 0, 6, and 12 h heat treated samples, respectively. Table S2 listed comprehensive information on all classes of sRNA sequence tags. 

To examine which type of sRNAs is associated with heat tolerance of flowering Chinese cabbage, Rfam database was searched to categorize these clean sequence reads into different classes (Table 1). rRNA reads were 0.36%–1.26% in HT genotypes and 0.69% in HS genotypes. tRNA read was higher (0.26%–5.38%) in HT genotypes than in HS genotypes (0.09%–0.16%). In addition, snRNA was 0.06%-0.12% for HT genotypes and 0.03%-0.07% for HS genotypes; snoRNA was 0.03% for both HT and HS genotypes. After removal of tRNA, rRNA, snRNA, and snoRNA, 22,222,402, 13,150,565, and 17,741,890 unique reads from the HT genotype and 25,021,953, 13,857,771, and 24,056,406 unique reads from the HS genotype treated with 0, 6, and 12 h under high temperature were mapped, respectively.

**Figure 1:**
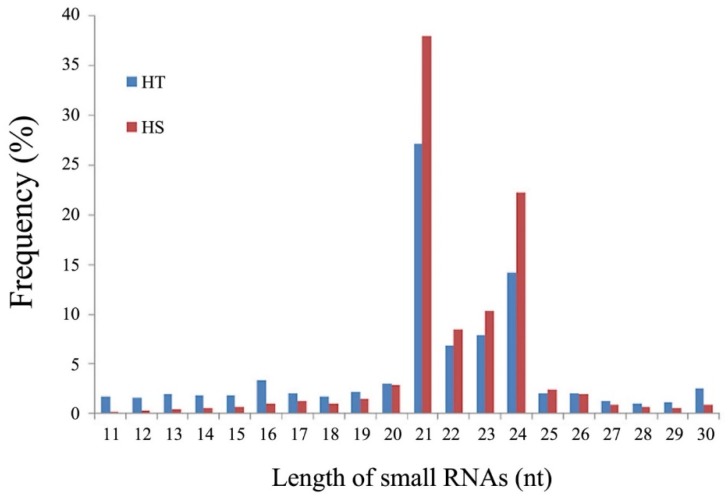
Sequence read length distribution of the small RNAs (sRNAs) from the heat-tolerant (HT) and heat-sensitive (HS) genotypes of flowering Chinese cabbage.

The sequence read length distribution patterns of sRNAs were similar between the HT and HS libraries in general, but a higher proportion of sRNAs were observed in the HS genotype (79.1%) than in the HT genotype (50.7%). The lengths of sRNAs were mainly from 21 to 24 nt (Figure 1) with the predominant length of 21 nt, followed by 24, 23, and 22 nt in both the HT and HS genotypes (Table S3).

### 3.2. Known miRNAs from Flowering Chinese Cabbage

To identify the known miRNAs from flowering Chinese cabbage, sRNA sequences were searched against the known miRNAs in miRBase (release 17.0) to find these reads with no more than three mismatched nucleotides as known miRNAs. The lengths of the known miRNAs usually ranged from 21–24 nt. Sixty-two small RNAs were identified to have identical sequences to *B. campestris* in miRBase and were considered as known bra-miRNAs (Table S4). Although many miRNAs had a relatively low expression, they showed significantly differential expression between HT and HS genotypes. The top ten abundantly expressed miRNAs were bra-miR398-3p, bra-miR168a-5p, bra-miR396-5p, bra-miR168b-5p, bra-miR171e, bra-miR160a-5p, bra-miR159a, bra-miR162-3p, bra-miR171a, and bra-miR156a-5p.

### 3.3. Identification of Novel miRNAs

**Figure 2:**
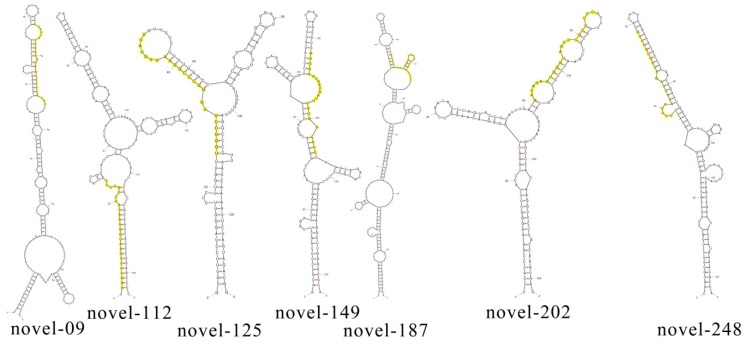
The predicted secondary structures of novel MicroRNAs (miRNA) precursors for seven novel miRNAs. Yellow color indicates mature miRNA.

Novel miRNA candidates were identified on the basis of MFE value, the miRNA/miRNA* duplex and secondary structure of precursor sequences. The stem-loop hairpin secondary structures were predicted from precursor sequences, suggesting that most of the identified miRNAs rely on the 5′ arm of the hairpin structure. To determine if novel miRNAs were involved in heat tolerance in flowering Chinese cabbage, all mappable sRNA sequences were searched against the *Brassica* database and the miRBase to eliminate previously known miRNAs. Any sRNAs that could be exactly mapped to the reference genome but not as conserved miRNAs were assumed to be novel miRNA candidates. To increase the accuracy of novel miRNA prediction, the miRNA/miRNA* criterion was evaluated. A total of 49 novel miRNA candidates were identified from the HT and HS libraries of flowering Chinese cabbage (Table S5). The mean MFE value predicted for pre-miRNAs was –42.13 kcal/mol, ranging from –23.6 to –217.2 kcal/mol. The identified novel miRNA length varied from 21 to 24 nt. Novel-mir09, novel-mir112, novel-mir125, novel-mir149, novel-mir187, novel-mir202, and novel-mir248 had striking secondary structures with lower MFE values and were considered as key putative miRNAs (Figure 2). Of all the novel miRNAs, novel-mir202, novel-mir225, novel-mir255, novel-mir248, novel-mir187, novel-mir170, and novel-mir99 had the highest expression levels.

### 3.4. Differential Expression Profiling of Known and Novel miRNAs

**Figure 3:**
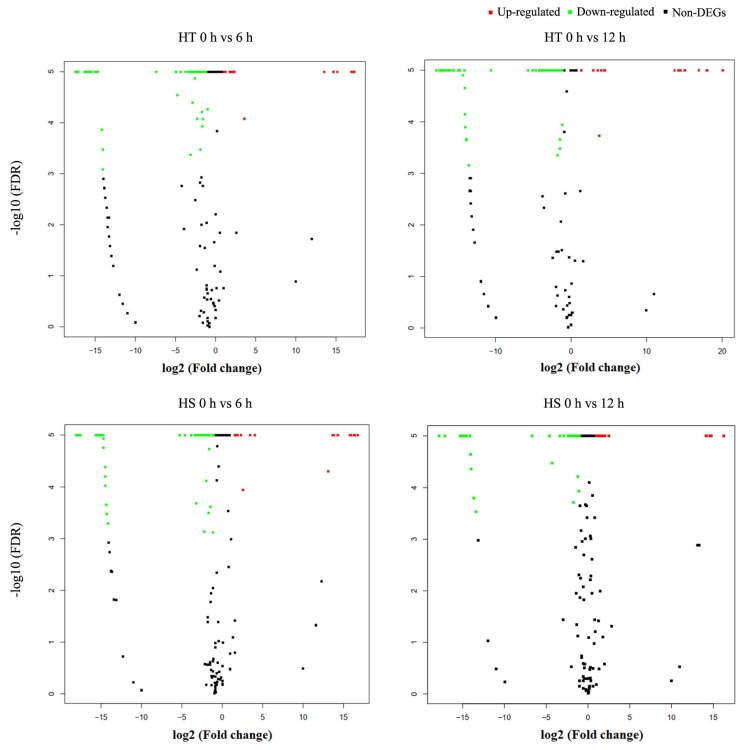
Differential expression of heat responsive miRNAs in heat-tolerant (HT) and heat-sensitive (HS) genotypes of flowering Chinese cabbage. Non-DEGs refer to miRNAs with non-differential expression.

To investigate the heat induced miRNAs in flowering Chinese cabbage, differentially expressed miRNAs were identified between heat treated (6 and 12 h heat treatments) and non-heat treated (0 h control) HT and HS genotypes (Figure 3). A total of 43 known and 49 novel miRNAs were differentially expressed in at least one of two time points (6 and 12 h) of heat treatments. 

Among the heat induced known miRNAs, 21 (4 up-regulated and 17 down-regulated) were differentially expressed in both HT and HS genotypes, whereas nine (1 up-regulated and 8 down-regulated) were differentially expressed only in the HT genotype (Table 2) and 13 (2 up-regulated and 11 down-regulated) were differentially expressed only in the HS genotype (Table S6). Among the novel miRNAs, 6 (1 up-regulated and 5 down-regulated) were differentially expressed in both HT and HS genotypes, 14 (3 up-regulated and 11 down-regulated) were differentially expressed in HT genotype only, and 29 (7 up-regulated and 22 down-regulated) were differentially expressed in HS genotype only (Table S7). The five differentially expressed known miRNAs and novel miRNAs that were common in both HT and HS genotypes are listed in Table 3. 

### 3.5. Putative Target Genes of Differentially Expressed miRNAs in HT and HS Genotypes

TAPIR [30] and TargetFinder [29] were used to identify the targets of the differentially expressed miRNAs under heat stress. Predicted target genes of heat responsive miRNAs included dihydrolipoyllysine-residue acetyltransferase component 1 of pyruvate dehydrogenase complex (novel-mir128), protein phloem protein 2-like A5-like (novel-mir243), protein phloem protein 2-like A8-like (novel-mir243), protein suppressor of npr1-1, constitutive 1-like (bra-miR1885b), flowering time control protein FCA (bra-miR824), interferon-induced guanylate-binding protein 2-like (bra-miR824), agamous-like MADS-box protein AGL16 (bra-miR824), IAA-alanine resistance protein 1-like (bra-miR400-5p), probable receptor-like protein kinase At5g47070 (bra-miR400-5p), pentatricopeptide repeat-containing protein At1g06580 (bra-miR400-5p), vacuolar protein sorting-associated protein 32 homolog 1-like (bra-miR396-3p), G-type lectin S-receptor-like serine/threonine-protein kinase RLK1 (bra-miR396-3p), transcriptional regulator *SUPERMAN* (bra-miR391-5p), reticuline oxidase-like protein (bra-miR391-5p), and several disease resistance proteins including TAO1-like (novel-mir243), RML1A-like isoform X1 (novel-mir243), At4g11170 (novel-mir243), *RPS6* (bra-miR1885b), and RPS6-like isoform X1 (bra-miR1885b) (Table 4; Table S8). These genes may play key roles in responses to heat-stress.

Targets of the differentially expressed miRNAs that are usually responsive to heat-stress include protein strictosidine synthase (novel-mir23), agamous-like MADS-box protein AGL17 isoform X1 (novel-mir23), protein NBR1 homolog (novel_mir99), *SEC12-like* protein 1 (novel-mir125), serine/threonine-protein kinase SRK2I (novel-mir187), probable N-acetyltransferase HLS1 (novel-mir202), serine hydroxymethyltransferase 7 (novel-mir214), protein detoxification 23 (novel-mir214), CBL-interacting serine/threonine-protein kinase 8 (bra-miR5718), protein ABC transporter 1 (bra-miR172c-3p, and bra-miR172d-3p), 1-aminocyclopropane-1-carboxylate synthase 9 (bra-miR398-5p), NAC domain-containing protein 92-like (bra-miR164b-5p), presenilin-like protein At1g08700 (bra-miR156a-3p), auxin response factor 16 (bra-miR160a-5p), heat shock 70 kDa protein 6 chloroplastic-like (bra-miR162-3p), and probable disease resistance protein At1g12290 isoform X2 (bra-miR172b-5p). Bra-miR1885, bra-miR824, bra-miR156, and bra-miR1885 are well-known for their putative functions in *B. campestris* ssp. *Chinensis* [24].

### 3.6. Functional Annotation of miRNA Target Genes

**Figure 4:**
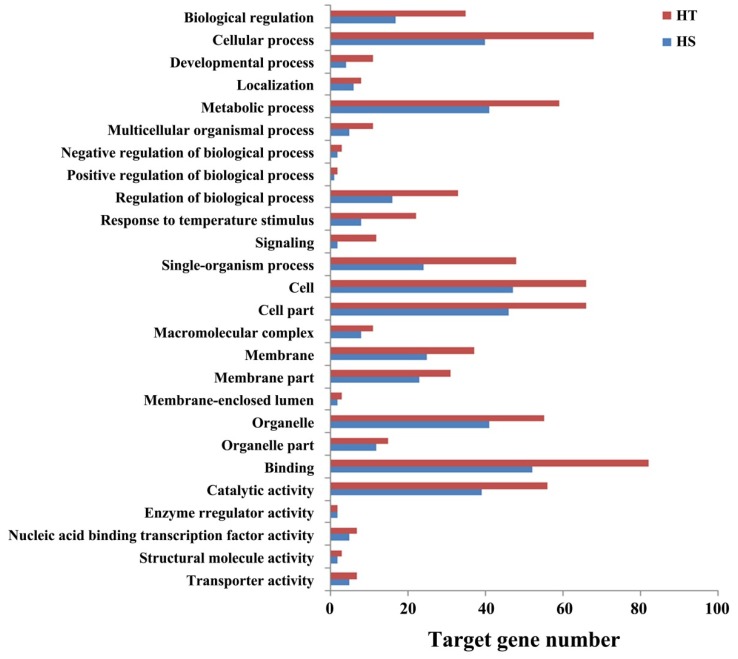
Gene Ontology (GO) annotation for target genes of heat responsive microRNAs in the heat-tolerant (HT) and heat-sensitive (HS) genotypes of flowering Chinese cabbage.

GO analysis on the putative miRNA target genes further divided them into three function categories: biological process, molecular function, and cellular component (Figure 4; Table S9). In the biological process category, the most enriched GO terms include biological regulation, cellular process, metabolic process, response to temperature stimulus, regulation of biological process, and single organism process. Interestingly, these target genes might play a significant role in diversifying biological processes such as signaling, localization, response to stimulus, and developmental process. In cellular component category, the most enriched GO terms include cell, cell part, membrane, macromolecular complex, organelle, and organelle part. The majority of molecular functions include nucleic acid binding transcription factor activity, catalytic activity, and transporter activity and binding.

**Figure 5:**
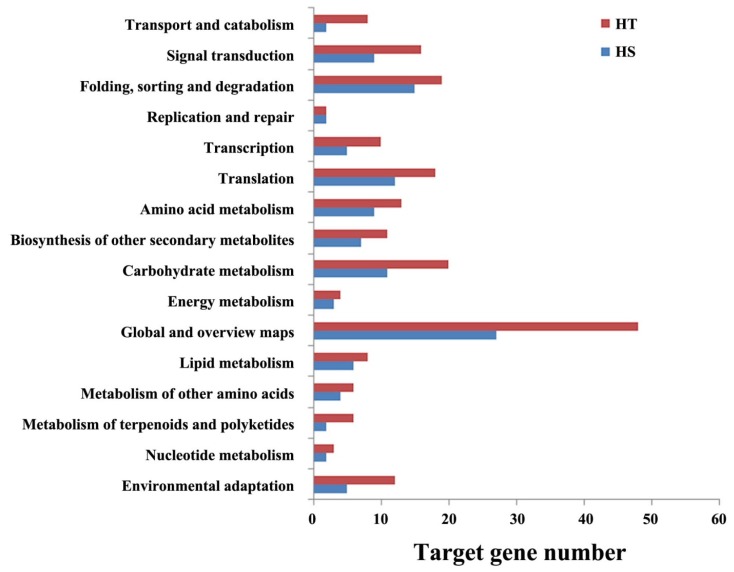
KEGG pathway analysis of target genes of heat responsive microRNAs in the heat-tolerant (HT) and heat-sensitive (HS) genotypes of flowering Chinese cabbage.

KEGG analysis predicted the potential pathway networks that were involved in response to heat stress in flowering Chinese cabbage including transport and catabolism, signal transduction, folding, sorting and degradation, environmental adaptation, replication and repair, transcription, and translation (Figure 5; Table S10).

The KEGG pathway analysis also predicted various miRNA-regulated pathways based on the miRNA targeted genes identified and demonstrated enriched target genes including genes for stress response, stress tolerance, and stress adaptation [32,35]. MEKK1 and SnRK2 were up-regulated and their expression might play a key role in responses to cold/salt stresses, pathogen infection, and drought tolerance in HT genotype. SUMM2 and WRKY33 were expressed in both HT and HS genotypes and might be involved in regulation of cell death defense response and camalexin synthesis, respectively (Figure S1). 

### 3.7. Validation of Differentially Expressed miRNAs by RT-qPCR

**Figure 6:**
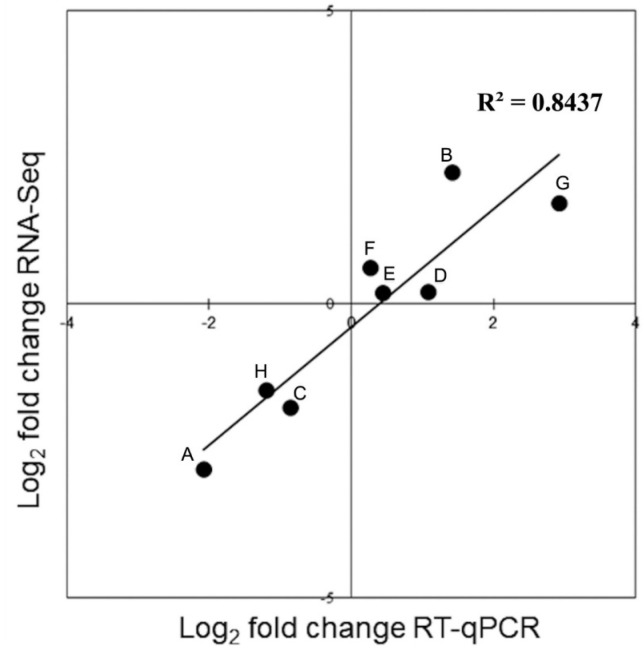
Validation of differentially expressed novel and known miRNAs using RT-qPCR. RNA at each time point was isolated from five biological replicates and mixed in an equivalent proportion for RT-qPCR analysis. Letters A to H represent the miRNA samples novel-mir23, novel-mir162, novel-mir214, novel-mir225, bra-miR5726, bra-miR160a-3p, bra-miR164e-5p, and bra-miR156e-3p, respectively.

To confirm the results from the RNA sequencing, four novel and four conserved miRNAs were randomly selected to represent both down- and up-regulated miRNAs for RT-qPCR. A strong correlation (r^2^ = 0.843) was detected between RT-qPCR and RNA-seq data (Figure 6), indicating a good agreement in expression levels (down-regulation or up-regulation) between RT-qPCR and RNA sequencing data for the selected miRNAs, confirming that the differentially expressed miRNAs of *B. campestris* ssp. *Chinensis* predicted by RNA sequencing were real.

## 4. Discussion

### 4.1. miRNA in Flowering Chinese Cabbage

Vegetable plants have to cope with diverse environmental stresses that may trigger numerous gene regulatory mechanisms, such as orchestration of gene expression regulation at a post-transcriptional level, and re-establishing and restoring cellular homeostasis, reducing cell-cycle regulation, and adaptive growth response [36,37]. Since the plant miRNA may involve in many functions in response to stress environments, it is an integral topic in functional genomic research. To date, numerous reports have revealed miRNA as key regulatory molecules that participate in plant metabolisms, stress responses, tissue development and many other functions [38,39]. The functional involvement of plant miRNAs in response to abiotic stresses was originally suggested by surveys of NCBI expressed sequence tags and profiling miRNA expression after challenging plants with certain stress stimuli to predict miRNA targets [40]. Flowering Chinese cabbage has much fewer miRNAs registered in miRBase compared to other vegetable crops and model plant Arabidopsis [11,41]. 

Using high-throughput sequencing, we identified 43 previously reported and 49 putative novel miRNAs in the HT and HS genotypes of flowering Chinese cabbage after heat treatments. sRNAs showed wide variation in sequence length, with 21 to 24 nt as most abundant, which agree with several previous reports [11,18]. Previously, we identified 41 conserved and 18 novel miRNAs in a flowering Chinese cabbage, Youlv 501, after heat treatment using high-throughput sequencing [21]. Likewise, using computational and deep sequencing methods, 24 novel and 161 known miRNAs from 51 families were identified in HT and HS genotypes of *Brassica oleracea* L. var *italic* [11]. High-throughput sequencing has been successfully used to identify novel and known miRNAs in response to stress in various species including soybean [42], maize [43], *Populus euphratica* [44], and rice [45]. In *B.* *rapa,* 21 novel miRNAs belonging to 19 miRNA families that were identified using NGS, bra-miR1885b.3 and bra-miR5718 were reported to be involved in response to heat [18]. Likewise, 221 conserved and 125 novel miRNAs were reported to play key functions in plant development and growth, metabolism, and stress responses in *B. rapa* [46]. Using a comparative genomics approach, 126 novel miRNAs were identified in *B. juncea* and were predicted to participate in regulation of different biological processes in response to drought, salinity, and high temperature stresses [20]. These findings reveal that more novel miRNAs with important regulatory functions in flowering Chinese cabbage can be identified using improved reference genome sequences.

### 4.2. miRNAs Are Involved in Heat Stress Responses 

Plant growth and development are highly affected by various types of stresses [47,48,49,50]. miRNAs in plants possess multiple mechanisms to respond to different stresses in tissues. Heat stress may induce different metabolic pathways and uncouple enzymes to add excess reactive oxygen species (ROS). To survive under high temperature, plants may use cellular antioxidant defense systems to defend vegetable crops from heat stress [51,52]. In the current study, bra-miR1885a, bra-miR5718, bra-miR5726, bra-miR160a, bra-miR172c-3p, bra-miR390-5p, and bra-miR400-5p were significantly up-regulated and bra-miR157a, bra-miR398-5p, bra-miR5719, bra-miR156e-3p, bra-miR400-5p were significantly down-regulated in the HT and HS flowering Chinese cabbage genotypes. In a previous report, bra-miR9557-3p, bra-miR160a-5p, bra-miR390-3p, bra-miR164a, bra-miR158-5p, and bra-miR156a-3p were downregulated and bra-miR5725, bra-miR159a, miR172c-3p, and bra-miR5726 were up-regulated in Youlv 501 under heat stress [21]. In addition, differentially expressed bra-miR391-3p, bra-miR9408-3p, bra-miR159a, bra-miR5712, bra-miR1140, bra-miR158-3p, bra-miR390-3p, and Novel-mir013 were reported as the known miRNAs in Youlv 501 [21], which were also identified in the current study. miR827, miR156h, and miR156g, miR5718, bra-miR1885b.3, and bra-miR571 were reported to be specifically up/down-regulated under heat stress in *Brassica* plants [18,53]. In a HT *Brassica* genotype, Catalase 2 enzymes reduced *HSP* expression, which in turn detoxified heat-induced ROS [11]. miR827 directly targeted to Catalase 2 gene in broccoli [18] and Arabidopsis [54]. *bol*-novel-09 and *bol*-novel-39 targeted different proteins that played an important role in heat-induced oxidative stress in *Arabidopsis* [55]. Yu et al. [18] reported that transgenic plants that overexpressed bra-miR5718, bra-miR398, and bra-miR1885b.3 showed improved thermotolerance. miR156 has been reported to be specifically induced under heat stress in broccoli [18] and wheat [56]. Moreover, after summarizing the results from various species, Zhang et al. [57] found that miR159, miR396, miR399, miR159, miR393, miR156, and miR395 were heat-stress related miRNAs. All these findings indicate that miRNAs play important roles in regulating heat stress in different species.

### 4.3. miRNA–Targeted Gene-Networks Involved in Response of Flowering Chinese Cabbage to Heat Stress

Upon heat stress, several genes that are responsive to abiotic stress in plants can be directly targeted by miRNAs. Target mRNA cleavage depends upon its complementary miRNA. It has been shown that conserved miRNA miR398a targeted *BracCSD1* gene, and there is an inverse relationship between miRNA and its target in *Brassica rapa* [18], indicating that the gene is heat-sensitive and the miRNA exhibits a heat-inhibitive function in response to heat stress. Furthermore, it has been revealed that several plants used more phosphate and nitrogen under heat stress than normal growth conditions, and this phenomena may be due to miRNA repression and roles of the target genes in adaptation and regulation of Pi starvation [58]. Moreover, miR156h and miR156g targeted *SPL* -family genes that were involved in floral transition and vegetative phase of *B. rapa* [19], and miR156a regulated *SPL2*, *SPL3*, *SPL9*, and *SPL10* targets, but *SPL2* showed a significant down-regulation under heat stress.

In our study, bra-miR156a-5p and 14 other miRNAs were involved in different biological functions (Table 4 and Table S8). We previously identified 432 potential mRNA targets for both conserved and novel miRNAs in Youlv 501 under heat stress. The identified targets were involved in regulation of the key functions including abiotic stress responses, cell, cell parts, cellular processes, and catalytic activity [21]. Meanwhile, miR395a, miR169f, miR827, bol-novel-26, miR169f, miR156b, bol-novel-01, and bol-novel-03 were significantly up-regulated between HT and HS genotypes [11]. It is suggested that numerous miRNAs play key roles in increasing head-forming capacity through regulation of molecular mechanisms of thermotolerance by targeting several genes including miR156b (*SPL9*), miR169f (*NF-YA1*), miR827 (*CAT2*), miR395a (*APS1*), miR172d (*TOE1*), bol-novel-01 (*PAPS2*), and bol-novel-03 (*ARF1*) [11]. Furthermore, miR169f targeted the AtNF-YA transcription factor and miR172 putatively targeted APETALA2 (AP2)-like family of transcription factors such as *TOE2*, *AP2*, *SCHLAFMÜTZE* (*SMZ*), and *TOE1*, and these genes play a significant role in flowering and maintaining floral meristem size in broccoli [11]. In our study, 14 novel and nine known miRNA were differentially expressed only in the heat-tolerant genotype under heat-stress, therefore, their target genes including disease resistance protein TAO1-like, *RPS6,* reticuline oxidase-like protein, etc. may play important roles in enhancing heat-tolerance. Likewise, miRNAs targeted the disease resistance protein RPS6 in soybean [59]. In our study, bra-miR172 putatively targeted AP2-like transcription factors including floral homeotic protein *APETALA 2*, AP2-like ethylene-responsive transcription factor *TOE2*, and *TOE3* under heat stress in flowering Chinese cabbage. Moreover, numerous miRNAs targeted transcription factors, including those disease resistance protein TAO1 (bra-miR5719), transcription factor TCP (bra-miR319-3p), probable N-acetyltransferase HLS1 (novel-mir202), squamosa promoter binding proteins (bra-miR156/ bra-miR157), and serine/threonine-protein kinase SRK2I (novel-mir187). Likewise, bol-novel-34 (phosphatase 2C), miR395 (ATP sulfurylases), and bol-novel-26 (auxin response factor 1) were involved in mediating the thermotolerance mechanisms in broccoli [19]. 

In conclusion, in this extensive study, we analyzed heat-responsive miRNAs from HT and HS genotypes of flowering Chinese cabbage after heat stress treatments and identified 49 novel and 43 known miRNAs that possess important regulatory roles in regulating heat stress responses by targeting numerous genes. The relationships between the target genes and the miRNAs in response to heat stress revealed from this study could help us design new breeding tools using biotechnological approaches for genetic improvement of heat tolerance of flowering Chinese cabbage.

## Figures and Tables

**Table 1:** Classification of heat-responsive small RNAs of heat tolerant (HT) and heat susceptible (HS) genotypes of flowering Chinese cabbage.

Read Type	HT			HS		
	0 h	6 h	12 h	0 h	6 h	12 h
Total	25,331,960 (100%)	22,632,118 (100%)	19,999,279 (100%)	27,647,469 (100%)	26,043,571 (100%)	26,275,173 (100%)
Intergenic	15,914,365 (62.82%)	7,884,837 (34.84%)	14,061,923 (70.31%)	15,559,731 (56.28%)	9,035,253 (34.69%)	12,686,730 (48.28%)
Intron	1,074,553 (4.24%)	420,049 (1.86%)	423,989 (2.12%)	939,369 (3.4%)	560,265 (2.15%)	894,981 (3.41%)
Exon	794,907 (3.14%)	580,063 (2.56%)	318,139 (1.59%)	842,483 (3.05%)	650,488 (2.5%)	1,283,163 (4.88%)
Precursor	144,519 (0.57%)	249,867 (1.1%)	137,914 (0.69%)	198,968 (0.72%)	103,036 (0.4%)	176,153 (0.67%)
Mature	4,129,468 (16.3%)	3,858,651 (17.05%)	2,127,450 (10.64%)	7,304,825 (26.42%)	3,398,482 (13.05%)	8,806,566 (33.52%)
Rfam other sncRNA	4412 (0.02%)	15,136 (0.07%)	81,044 (0.41%)	5515 (0.02%)	7588 (0.03%)	6884 (0.03%)
rRNA	98,508 (0.39%)	112,095 (0.5%)	252,496 (1.26%)	148,003 (0.54%)	83,184 (0.32%)	180,019 (0.69%)
snRNA	16,377 (0.06%)	27,407 (0.12%)	11,777 (0.06%)	13,109 (0.05%)	8068 (0.03%)	18,387 (0.07%)
snoRNA	7442 (0.03%)	6920 (0.03%)	6907 (0.03%)	5794 (0.02%)	4392 (0.02%)	7306 (0.03%)
tRNA	65,593 (0.26%)	22,894 (0.1%)	1,076,247 (5.38%)	43,977 (0.16%)	27,661 (0.11%)	24,478 (0.09%)
Unmapped	3,081,816 (12.17%)	9,454,199 (41.77%)	1,501,393 (7.51%)	2,585,695 (9.35%)	12,165,154 (46.71%)	2,190,506 (8.34%)

**Table 2:** Differentially expressed heat responsive novel and known miRNAs identified in heat tolerant (HT) flowering Chinese cabbage.

miRNA Name	Sequence (5>3)	HT Count (0 h)	HT Count (6 h)	log_2_ Ratio (HT 6/HT 0)	*p*-Value	HT Count (0 h)	HT Count (12 h)	log_2_ Ratio (HT 12/HT 0)	*p*-Value
**Novel miRNAs**									
novel_mir27	TCAAGCTGGTGTCTGGATGAGT	155	47	−1.71	3.28 × 10^−5^	155	0	−17.23	6.96 × 10^−41^
novel_mir95	GGTGAGGTCGCTCTGAGAGGATAG	29	0	−14.8	1.89 × 10^−6^	29	0	−14.77	3.32 × 10^−8^
novel_mir149	GGGGAACGACGATTTGTGACACC	143	0	−17.1	2.41 × 10^−29^	143	29	−2.25	7.67 × 10^−15^
novel_mir46	GAAAACTATTCGATACATATGGCC					0	15	13.66	5.58 × 10^−6^
novel_mir89	AGGGACAGAGGACTGACATGTGGC					107	0	−16.62	1.96 × 10^−28^
novel_mir105	ACTAAATCTACACCAATATTGAT					85	0	−16.29	9.93 × 10^−23^
novel_mir114	ATTCTTGAGTCCTTAATACATATA					20	0	−14.29	7.16 × 10^−6^
novel_mir120	GACTCTAAAAATACCCTTGGTACTT					79	21	−1.85	6.84 × 10^−7^
novel_mir177	TATTCCCGCGAAACCCACGGC	0	13	13.55	1.82 × 10^−6^				
novel_mir238	CCTGCGGCTGCGGCGATATT					241	0	−17.86	3.46 × 10^−63^
novel_mir240	CAATGGGATCCGCGAACAGTGCA					17	0	−14.05	4.29 × 10^−5^
novel_mir243	GCTGATGGAACACTGGCCCGGCCCA					0	20	14.21	1.03 × 10^−7^
novel_mir250	TATAGTTAGGCGTTAGGCACTATG					104	0	−16.67	1.17 × 10^−27^
novel_mir255	CAAGCGGTTCAACTGCGGTGCGGT	1494	494	−1.67	5.79 × 10^−31^				
**Known miRNAs**									
bra-miR156e-3p	TGCTCACCTCTCTTTCTGTCAGT					1971	696	−1.51	6.29 × 10^−90^
bra-miR824	TAGACCATTTGTGAGAAGGGA	1026	396	−1.39	2.77 × 10^−13^	1026	261	−2.01	7.86 × 10^−77^
bra-miR1885a	CATCAATGAAAGGTATGATTCC	1271	396	−1.68	4.82 × 10^−31^				
bra-miR1885b	TACATCTTCTCCGCGGAAGCTC	1546	572	−1.42	1.63 × 10^−22^				
bra-miR172d-5p	GCAGCATCATTAAGATTCACA					1	14	3.70	0.00011
bra-miR400-5p	TATGAGAGTATTATAAGTCAC	750	231	−1.7	1.77 × 10^−19^				
bra-miR396-3p	GCTCAAGAAAGCTGTGGGAAA	1694	693	−1.29	2.00 × 10^−16^				
bra-miR391-5p	TTCGCAGGAGAGATAGCGCCA	335	110	−1.59	3.48 × 10^−8^				
bra-miR2111b-3p	ATCCTCGGGATACGGATTACC	30	1	−4.75	1.48 × 10^−5^	30	0	−14.72	1.82 × 10^−8^

Missing values refer to that differentially expressed miRNAs were not significant between this heat treatment time point and control (0 h).

**Table 3:** Heat-induced differentially expressed known and novel miRNAs identified from both heat tolerant (HT) and heat-susceptible (HS) flowering Chinese cabbage genotypes.

miRNA Name	Sequence (5>3)	HT Count (6 h)	HS Count (6 h)	log_2_ Ratio (HT 6/HS 6)	*p*-Value	HT Count (12 h)	HS Count (12 h)	log_2_ Ratio (HT 12/HS 12)	*p*-Value
bra-miR156a-3p	GCTTACTCTCTCTCTGTCACC	39	92	−1.21	3.5 × 10^−6^	26	111	−2.09	3.02 × 10^−9^
bra-miR164e-3p	CACGTGCTCCCCTCCTCCAAC	15	0	13.66	1.85 × 10^−5^	14	0	13.77	6.52 × 10^−6^
bra-miR164e-5p	TGGAGAAGCAGGGCACGTGCAA	4	49	−3.61	4.42 × 10^−11^	36	11	1.66	3.41 × 10^−6^
bra-miR390-5p	AAGCTCAGGAGGGATAGCGCC	130	63	1.02	1.26 × 10^−7^	577	164	1.77	4 × 10^−83^
bra-miR5712	AATATTAATATAATTGGTGAG	18	71	−1.97	4.17 × 10^−8^	95	477	−2.40	1.41 × 10^−41^
novel_mir23	ACCCGTCCATGGGCCCCAGGCTCA	0	37	−15.13	2.31 × 10^−11^	26	182	−2.79	1.98 × 10^−16^
novel_mir112	AGGCTCCGAATGGTAACATCCGTCCC	97	0	16.55	2.93 × 10^−31^	92	477	−2.37	1.38 × 10^−30^
novel_mir128	AATTAAGAAACTCCCATTGGACCGC	0	16	−13.87	2.56 × 10^−5^	24	79	−1.72	3.31 × 10^−5^
novel_mir134	ACGTGGAACACTCTGACTAGTCTGAC	21	0	14.35	2.39 × 10^−7^	0	97	−16.56	2.1 × 10^−24^
novel_mir225	CCTGCGGCTGCGGCGATATT	56	13	1.97	2.52 × 10^−8^	409	1569	−1.96	9.67 × 10^−98^

**Table 4:** Potential targets of differentially expressed novel miRNAs in flowering Chinese cabbage genotypes under heat stress.

miRNA	Target Name	Target Id	E-Value	Putative Function of Target
**Target genes of heat responsive miRNAs in the heat-tolerant genotype**				
novel-mir128	BraA09g043410.3C	XP_018458905.1	1.40E-77	Dihydrolipoyllysine-residue acetyltransferase component 1 of pyruvate dehydrogenase complex
novel-mir243	BraA09g015030.3C	XP_013688447.1	2.10E-291	Disease resistance protein RML1A-like isoform X1
novel-mir243	BraA02g007610.3C	XP_013616826.1	4.5E-53	Disease resistance protein TAO1-like
novel-mir243	BraA02g036020.3C	XP_009129096.1	2.2E-82	Protein PHLOEM PROTEIN 2-LIKE A5-like
novel-mir243	BraA06g012120.3C	XP_009149053.1	1.30E-195	Protein PHLOEM PROTEIN 2-LIKE A8-like
novel-mir243	BraA09g016880.3C	XP_013659354.1	4.20E-87	Putative disease resistance protein At4g11170
**Target genes of general heat-stress responsive miRNAs that were differentially expressed between HT and HS genotypes**				
novel-mir23	BraA01g022410.3C	XP_018508806.1	1.30E-183	Protein strictosidine synthase
novel-mir78	BraA05g033150.3C	XP_009146435.1	1.60E-77	Uncharacterized protein LOC103870086
novel-mir78	BraA05g027310.3C	XP_009145697.1	8.20E-247	Uncharacterized protein LOC103869376
novel_mir99	BraA08g020340.3C	XP_009109238.1	1.9E-272	Protein NBR1 homolog
novel-mir125	BraA04g007130.3C	KHN00936.1	1.60E-250	SEC12-like protein 1
novel-mir151	BraA10g012290.3C	XP_013639528.1	7.70E-54	Uncharacterized protein LOC106344762
novel-mir187	BraA07g017040.3C	XP_009103473.1	4.80E-200	Serine/threonine-protein kinase SRK2I
novel-mir202	BraA01g001310.3C	XP_013683960.1	7.40E-229	Probable N-acetyltransferase HLS1
novel-mir214	BraA08g005730.3C	XP_013605618.1	3.20E-27	Serine hydroxymethyltransferase 7
novel-mir214	BraA09g031950.3C	XP_009123743.1	1.30E-280	Protein DETOXIFICATION 23
novel-mir255	BraA08g009450.3C	NP_198334.1	3.30E-260	Purple acid phosphatase 26

## Supplementary Materials

The following are available online at https://www.mdpi.com/2073-4425/11/3/264/s1, Figure S1: KEGG pathway map for MAPK signaling for heat-tolerant (HT) and heat-sensitive (HS) genotypes of flowering Chinese cabbage; Table S1: List of primers used in this study; Table S2: Analysis of small RNA sequences from the libraries of HT and HS flowering Chinese cabbage; Table S3: The length distribution and percentage of detected reads from heat-responsive total small RNAs of the flowering Chinese cabbage HT and HS genotype by sequencing; Table S4: Expression profiles of heat-responsive conserved miRNAs in HT and HS genotypes of flowering Chinese cabbage at 6 h and 12 h; Table S5: Novel miRNAs identified from HT and HS flowering Chinese cabbage genotypes under heat stress; Table S6: Differentially expressed heat-responsive known miRNAs in flowering Chinese cabbage HT and HS genotypes at 6 h and 12 h; Table S7. Differentially expressed heat-responsive novel miRNAs in flowering Chinese cabbage HT and HS genotypes at 6 h and 12 h; Table S8: Potential targets of differentially expressed miRNAs between HT and HS Flowering Chinese cabbage under heat stress; Table S9; Gene Ontology (GO) annotation of target genes of heat stress-responsive microRNA in the HT and HS libraries of flowering Chinese cabbage; Table S10; KEGG classification of target genes of heat stress-responsive microRNAs in the HT and HS libraries of flowering Chinese cabbage.

## References

[1] Chen J., Li R., Xia Y., Bai G., Guo P., Wang Z., Zhang H., Siddique K.H. (2017). Development of EST-SSR markers in flowering Chinese cabbage (*Brassica campestris* L. ssp. *chinensis* var. *utilis* Tsen et Lee) based on de novo transcriptomic assemblies. PLoS ONE.

[2] Ahmed W., Xia Y., Li R., Bai G., Siddique K.H., Guo P. (2020). Non-coding RNAs: Functional roles in the regulation of stress response in *Brassica* crops. Genomics.

[3] Dresselhaus T., Hückelhoven R. (2018). Biotic and Abiotic Stress Responses in Crop Plants.

[4] Young L.W., Wilen R.W., Bonham-Smith P.C. (2004). High temperature stress of *Brassica napus* during flowering reduces micro-and megagametophyte fertility, induces fruit abortion, and disrupts seed production. J. Exp. Bot..

[5] Bita C., Gerats T. (2013). Plant tolerance to high temperature in a changing environment: Scientific fundamentals and production of heat stress-tolerant crops. Front. Plant. Sci..

[6] Battisti D.S., Naylor R.L. (2009). Historical warnings of future food insecurity with unprecedented seasonal heat. Science.

[7] Wang W., Vinocur B., Shoseyov O., Altman A. (2004). Role of plant heat-shock proteins and molecular chaperones in the abiotic stress response. Trend. Plant. Sci..

[8] Zhai Z., Lin Z., Chen H., Chen Z., Center G. (2016). Temporal and spatial variation of temperature suitability index for *Brassica parachinesis* in Guangdong. Guangdong Agric. Sci..

[9] Sato S., Katoh N., Iwai S., Hagimori M. (2002). Effect of low temperature pretreatment of buds or inflorescence on isolated microspore culture in *Brassica rapa* (syn. *B. campestris*). Breed. Sci..

[10] Kalisz A., Siwek P. (2006). Yield and quality of spring Chinese cabbage as affected by different temperature conditions during seedling production. Folia Horticul..

[11] Chen C.-C., Fu S.-F., Norikazu M., Yang Y.-W., Liu Y.-J., Ikeo K., Gojobori T., Huang H.-J. (2015). Comparative miRNAs analysis of two contrasting broccoli inbred lines with divergent head-forming capacity under temperature stress. BMC Genom..

[12] Voinnet O. (2009). Origin, biogenesis, and activity of plant microRNAs. Cell.

[13] Katiyar-Agarwal S., Jin H. (2010). Role of small RNAs in host-microbe interactions. Ann. Rev. Phyto..

[14] Larkindale J., Hall J.D., Knight M.R., Vierling E. (2005). Heat stress phenotypes of Arabidopsis mutants implicate multiple signaling pathways in the acquisition of thermotolerance. Plant. Physiol..

[15] Geng M., Li H., Jin C., Liu Q., Chen C., Song W., Wang C. (2014). Genome-wide identification and characterization of miRNAs in the hypocotyl and cotyledon of cauliflower (*Brassica oleracea* L. var. *botrytis*) seedlings. Planta.

[16] Wei L., Xiao M., Hayward A., Fu D. (2013). Applications and challenges of next-generation sequencing in *Brassica* species. Planta.

[17] Kozomara A., Griffiths-Jones S. (2013). miRBase: Annotating high confidence microRNAs using deep sequencing data. Nucleic Acids. Res..

[18] Yu X., Wang H., Lu Y., De Ruiter M., Cariaso M., Prins M., Van Tunen A., He Y. (2011). Identification of conserved and novel microRNAs that are responsive to heat stress in *Brassica rapa*. J. Exp. Bot..

[19] Wang A., Hu J., Huang X., Li X., Zhou G., Yan Z. (2016). Comparative transcriptome analysis reveals heat-responsive genes in Chinese cabbage (*Brassica rapa* ssp. chinensis). Front. Plant Sci..

[20] Bhardwaj A.R., Joshi G., Pandey R., Kukreja B., Goel S., Jagannath A., Kumar A., Katiyar-Agarwal S., Agarwal M. (2014). A genome-wide perspective of miRNAome in response to high temperature, salinity and drought stresses in *Brassica juncea* (Czern) L.. PLoS ONE.

[21] Ahmed W., Xia Y., Zhang H., Li R., Bai G., Siddique K.H., Guo P. (2019). Identification of conserved and novel miRNAs responsive to heat stress in flowering Chinese cabbage using high-throughput sequencing. Sci. Rep..

[22] Lee S.I., Muthusamy M., Nawaz M.A., Hong J.K., Lim M.-H., Kim J.A., Jeong M.-J. (2019). Genome-wide analysis of spatiotemporal gene expression patterns during floral organ development in *Brassica rapa*. Mol. Genet. Genom..

[23] Rehman H.M., Nawaz M.A., Shah Z.H., Ludwig-Müller J., Chung G., Ahmad M.Q., Yang S.H., Lee S.I. (2018). Comparative genomic and transcriptomic analyses of Family-1 UDP glycosyltransferase in three *Brassica* species and *Arabidopsis* indicates stress-responsive regulation. Sci. Rep..

[24] Jiang J., Lv M., Liang Y., Ma Z., Cao J. (2014). Identification of novel and conserved miRNAs involved in pollen development in *Brassica campestris* ssp. *chinensis* by high-throughput sequencing and degradome analysis. BMC Genom..

[25] Körbes A.P., Machado R.D., Guzman F., Almerao M.P., De Oliveira L.F.V., Loss-Morais G., Turchetto-Zolet A.C., Cagliari A., Dos Santos Maraschin F., Margis-Pinheiro M. (2012). Identifying conserved and novel microRNAs in developing seeds of *Brassica napus* using deep sequencing. PLoS ONE.

[26] Langmead B., Salzberg S.L. (2012). Fast gapped-read alignment with Bowtie 2. Nat. Method..

[27] Zuker M. (2003). Mfold web server for nucleic acid folding and hybridization prediction. Nucleic Acid. Res..

[28] Meyers B.C., Axtell M.J., Bartel B., Bartel D.P., Baulcombe D., Bowman J.L., Cao X., Carrington J.C., Chen X., Green P.J. (2008). Criteria for annotation of plant MicroRNAs. Plant Cell..

[29] Xie F., Xiao P., Chen D., Xu L., Zhang B. (2012). miRDeepFinder: A miRNA analysis tool for deep sequencing of plant small RNAs. Plant Mol. Biol..

[30] Bonnet E., He Y., Billiau K., Van de Peer Y. (2010). TAPIR, a web server for the prediction of plant microRNA targets, including target mimics. Bioinformatics.

[31] Srivastava P.K., Moturu T.R., Pandey P., Baldwin I.T., Pandey S.P. (2014). A comparison of performance of plant miRNA target prediction tools and the characterization of features for genome-wide target prediction. BMC Genom..

[32] Kanehisa M., Goto S. (2000). KEGG: Kyoto encyclopedia of genes and genomes. Nucleic Acid. Res..

[33] Shen Y., Zhang Z., Lin H., Liu H., Chen J., Peng H., Cao M., Rong T., Pan G. (2011). Cytoplasmic male sterility-regulated novel microRNAs from maize. Funct. Integr. Genom..

[34] Livak K.J., Schmittgen T.D. (2001). Analysis of relative gene expression data using real-time quantitative PCR and the 2− ΔΔCT method. Methods.

[35] Kulik A., Wawer I., Krzywińska E., Bucholc M., Dobrowolska G. (2011). SnRK2 protein kinases—key regulators of plant response to abiotic stresses. OMICS J. Integr. Biol..

[36] Tian Y., Tian Y., Luo X., Zhou T., Huang Z., Liu Y., Qiu Y., Hou B., Sun D., Deng H. (2014). Identification and characterization of microRNAs related to salt stress in broccoli, using high-throughput sequencing and bioinformatics analysis. BMC Plant Biol..

[37] Xiong L., Schumaker K.S., Zhu J.-K. (2002). Cell signaling during cold, drought, and salt stress. Plant Cell..

[38] Sunkar R., Li Y.-F., Jagadeeswaran G. (2012). Functions of microRNAs in plant stress responses. Trend. Plant. Sci..

[39] Sun X., Lin L., Sui N. (2019). Regulation mechanism of microRNA in plant response to abiotic stress and breeding. Mol. Biol. Rep..

[40] Hou J., Lu D., Mason A.S., Li B., Xiao M., An S., Fu D. (2019). Non-coding RNAs and transposable elements in plant genomes: Emergence, regulatory mechanisms and roles in plant development and stress responses. Planta.

[41] Santos L.S., Maximiano M.R., Megias E., Pappas M., Ribeiro S.G., Mehta A. (2019). Quantitative expression of microRNAs in *Brassica oleracea* infected with *Xanthomonas campestris* pv. campestris. Mol. Biol. Rep..

[42] Zeng Q.-Y., Yang C.-Y., Ma Q.-B., Li X.-P., Dong W.-W., Nian H. (2012). Identification of wild soybean miRNAs and their target genes responsive to aluminum stress. BMC Plant. Biol..

[43] Zhao M., Tai H., Sun S., Zhang F., Xu Y., Li W.-X. (2012). Cloning and characterization of maize miRNAs involved in responses to nitrogen deficiency. PLoS ONE.

[44] Li B., Qin Y., Duan H., Yin W., Xia X. (2011). Genome-wide characterization of new and drought stress responsive microRNAs in *Populus euphratica*. J. Exp. Bot..

[45] Heisel S.E., Zhang Y., Allen E., Guo L., Reynolds T.L., Yang X., Kovalic D., Roberts J.K. (2008). Characterization of unique small RNA populations from rice grain. PLoS ONE.

[46] Wang J., Yang X., Xu H., Chi X., Zhang M., Hou X. (2012). Identification and characterization of microRNAs and their target genes in *Brassica oleracea*. Gene.

[47] Pegler J.L., Oultram J.M., Grof C.P., Eamens A.L. (2019). Profiling the abiotic stress responsive microRNA landscape of *Arabidopsis thaliana*. Plants.

[48] Gahlaut V., Baranwal V.K., Khurana P. (2018). miRNomes involved in imparting thermotolerance to crop plants. 3 Biotech.

[49] Tang W., He X., Qian L., Wang F., Zhang Z., Sun C., Lin L., Guan C. (2019). Comparative transcriptome analysis in oilseed rape (*Brassica napus*) reveals distinct gene expression details between nitrate and ammonium nutrition. Genes.

[50] Ali M.A., Azeem F., Nawaz M.A., Acet T., Abbas A., Imran Q.M., Shah K.H., Rehman H.M., Chung G., Yang S.H. (2018). Transcription factors WRKY11 and WRKY17 are involved in abiotic stress responses in *Arabidopsis*. J. Plant. Physiol..

[51] Song X., Li Y., Cao X., Qi Y. (2019). MicroRNAs and their regulatory roles in plant–environment interactions. Ann. Rev. Plant. Biol..

[52] Chen Z., Huo Q., Yang H., Jian H., Qu C., Lu K., Li J. (2019). Joint RNA-Seq and miRNA profiling analyses to reveal molecular mechanisms in regulating thickness of pod canopy in *Brassica napus*. Genes.

[53] Wang L., Yu X., Wang H., Lu Y.-Z., De Ruiter M., Prins M., He Y.-K. (2011). A novel class of heat-responsive small RNAs derived from the chloroplast genome of Chinese cabbage (*Brassica rapa*). BMC Genom..

[54] Queval G., Issakidis-Bourguet E., Hoeberichts F.A., Vandorpe M., Gakière B., Vanacker H., Miginiac-Maslow M., Van Breusegem F., Noctor G. (2007). Conditional oxidative stress responses in the Arabidopsis photorespiratory mutant *cat2* demonstrate that redox state is a key modulator of daylength-dependent gene expression, and define photoperiod as a crucial factor in the regulation of H_2_O_2_-induced cell death. Plant J..

[55] Cheng M.-C., Liao P.-M., Kuo W.-W., Lin T.-P. (2013). The Arabidopsis ETHYLENE RESPONSE FACTOR1 regulates abiotic stress-responsive gene expression by binding to different cis-acting elements in response to different stress signals. Plant Physiol..

[56] Wu L., Zhou H., Zhang Q., Zhang J., Ni F., Liu C., Qi Y. (2010). DNA methylation mediated by a microRNA pathway. Mol. Cell.

[57] Zhang B. (2015). MicroRNA: A new target for improving plant tolerance to abiotic stress. J. Exp. Bot..

[58] Fujii H., Chiou T.-J., Lin S.-I., Aung K., Zhu J.-K. (2005). A miRNA involved in phosphate-starvation response in *Arabidopsis*. Curr. Biol..

[59] Zhao M., Cai C., Zhai J., Lin F., Li L., Shreve J., Thimmapuram J., Hughes T.J., Meyers B.C., Ma J. (2015). Coordination of microRNAs, phasiRNAs, and NB-LRR genes in response to a plant pathogen: Insights from analyses of a set of soybean *Rps* gene near-isogenic lines. Plant. Genom..

