# Supplementary figures and images for: Comparative Analysis of miRNA Expression Profiles between Heat-Tolerant and Heat-Sensitive Genotypes of Flowering Chinese Cabbage Under Heat Stress Using High-Throughput Sequencing
Source: Genes (Basel). 2020 Feb 28;11(3):264. doi: 10.3390/genes11030264 (PMC7140848; doi:10.3390/genes11030264)

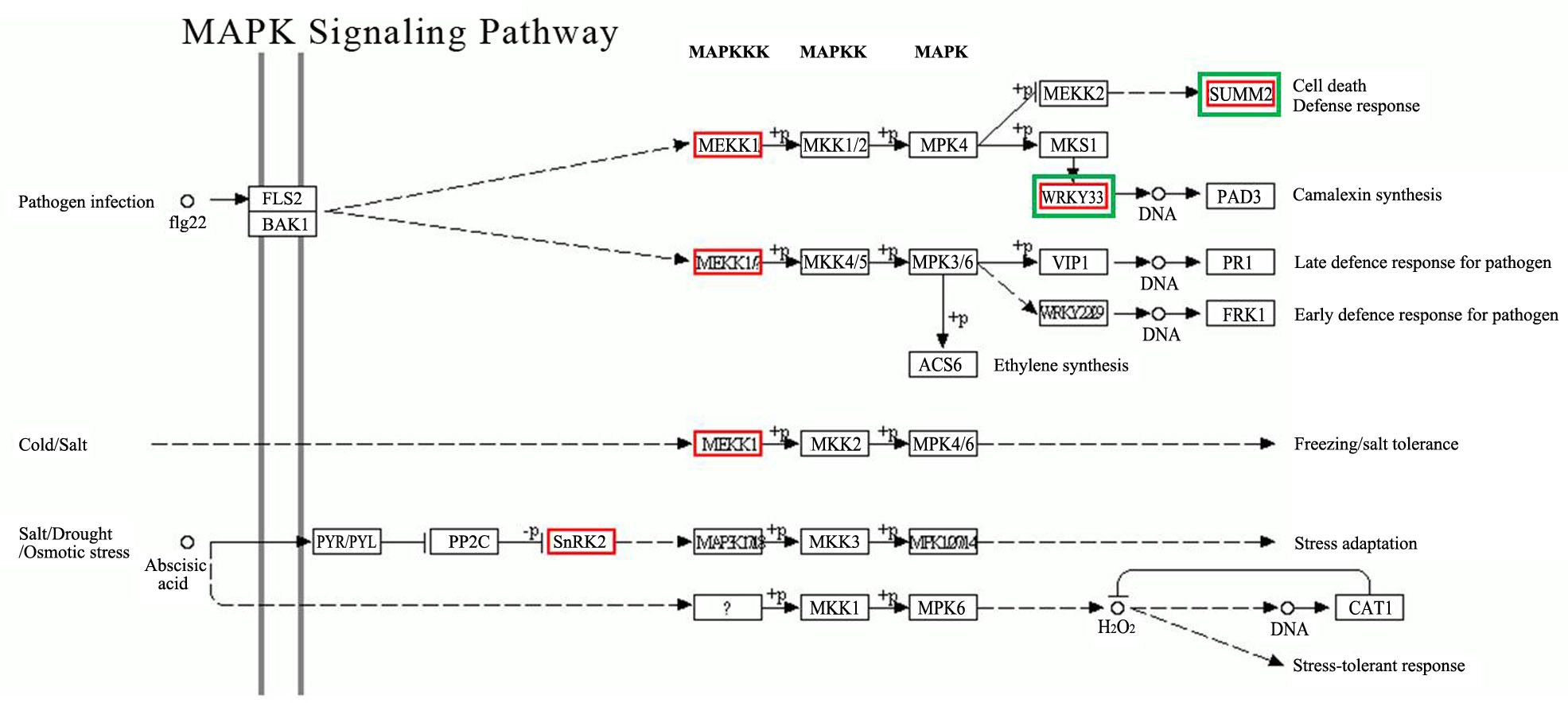

Supplement: Supplementary file 1 [file genes-11-00264-s001.zip › Figure S1.jpg]
